# Effects of *in vitro* fermentation of *Astragalus* polysaccharide on gut microbiota and neurotransmitter metabolites in patients with major depressive disorder

**DOI:** 10.3389/fpsyt.2026.1752933

**Published:** 2026-02-25

**Authors:** Chen Lin, Yu-Wei Mi, Huo-Wang Zheng, Yan-Bin Hou, Jie-Qiong Hu, Jia-Xin Mao, Ni Dai, Chao-Lang Fu, Xiao-Qiong Li, Yun-Xin Ji

**Affiliations:** 1 Department of Psychosomatic Medicine, The First Affiliated Hospital of Ningbo University, Ningbo, Zhejiang, China; 2 State Key Laboratory for Managing Biotic and Chemical Threats to the Quality and Safety of Agro-products, Zhejiang Academy of Agricultural Sciences, Hangzhou, Zhejiang, China; 3 Rehabilitation Department, The People’s Hospital of Ningbo Haishu District, Ningbo, Zhejiang, China

**Keywords:** Astragalus polysaccharide, depression, gamma-aminobutyric acid, gut microbiota, *in vitro* fermentation, metabolites

## Abstract

**Background:**

Major depressive disorder (MDD) is a common and heterogeneous mental illness with an unclear pathogenesis and often suboptimal treatment outcomes. *Astragalus* polysaccharide (APS) has shown potential antidepressant effects and may serve as a functional food; however, its impact on the gut microbiota and metabolome in MDD remains unexplored.

**Methods:**

Using an *in vitro* fermentation model coupled with 16S rRNA sequencing and targeted metabolomics, we characterized the gut microbial composition and neurotransmitter metabolites in fecal samples from 15 MDD patients and 15 healthy subjects.

**Results:**

MDD samples exhibited elevated *Fusobacteriaecae* and *Eggerthellaceae*, reduced levels of *Eubacterium hallii*, *Faecalibacterium*, and *Ruminococcus*, and higher concentrations of glutamine (Gln) and glutamate (Glu). APS fermentation significantly increased *Bifidobacterium* and *Lactobacillus* while decreasing *Eubacterium hallii* and *Bilophila*, without increasing the total gas volume, CO_2_, and H_2_S production. Additionally, beneficial metabolites were elevated in MDD samples after APS addition, including glutamine (Gln), γ-aminobutyric acid (GABA), dopamine (DA), and 5-hydroxytryptophan (5-HTP). The increase in GABA among healthy subjects was more significant.

**Conclusions:**

These findings indicate that MDD is associated with disruptions in gut microbiota and Glu/GABA metabolism. When APS are applied directly to the colon, they may mitigate these disruptions by modulating microbial composition and key neuroactive metabolites, thereby influencing the central nervous system via the gut-brain axis. This effect appears to be more pronounced in healthy individuals.

**Clinical Trial Registration:**

https://www.medicalresearch.org.cn, identifier MR-33-25-010733.

## 1 Introduction

Major depressive disorder (MDD) is a prevalent psychiatric disorder, affecting at least 350 million individuals globally, with a prevalence rate of approximately 6.8% in China (1), but the efficacy of commonly used antidepressants does not exceed 50% (2). Therefore, there is an urgent need to explore an effective treatments for MDD.

Previous research has demonstrated an enhanced understanding of the role of gut microbiota in the bidirectional communication mechanism of the “brain-gut” axis, leading to the establishment of the “microbiota-gut-brain axis (MGBA)” concept (3). This axis is crucial in the pathogenesis and progression of depression, primarily through the modulation of gut microbiota, their metabolites, and neurotransmitter levels (4). A growing body of evidence indicates that patients with MDD exhibit intestinal dysbiosis, characterized by an increased abundance of *Firmicutes*, *Actinomycetes*, and *Bacteroidetes*, alongside a reduction in *Bifidobacterium* and *Lactobacillus* populations (4, 5). In the chronic stress depression model, research has demonstrated that stress can disrupt the transmission and metabolism of neurotransmitters between the gut and the brain, resulting in elevated levels of glutamate (Glu) and subsequent functional impairments of gamma-aminobutyric acid (GABA). This disruption collectively influences the onset and progression of depression (6). Modulating the gut microenvironment has been shown to enhance neurotransmitter activity and mitigate depressive symptoms (7). One study found that *Bifidobacterium* and *Lactobacillus* can ferment to produce substantial amounts of GABA, which alleviates depression-related symptoms and intestinal dysfunction in mice. This suggests that alterations in the gut microenvironment and its metabolites play a significant role in the pathogenesis of MDD (8). Consequently, dysregulation of the gut microbiota and associated metabolites is anticipated to be a novel target for MDD treatment.

Recent investigations have extensively explored the interactions between traditional Chinese medicine (TCM) and intestinal microbiota, as well as metabolic processes. A growing body of evidence indicates that various TCM components, particularly polysaccharides, possess the capacity to regulate intestinal flora (9). These polysaccharides serve as fermentation substrates for human intestinal symbiotic bacteria, thereby influencing the growth and metabolic activities of the gut microbiota (10). *Astragalus*, a widely utilized medicinal and edible homologous material, has been documented to exhibit antidepressant properties (11). Among its constituents, *Astragalus* polysaccharide (APS) is the most prevalent and is recognized for its significant anti-inflammatory, antioxidant, immunomodulatory, neuroprotective, and antidepressant effects (12). APS has demonstrated efficacy in various disease models, including its ability to modulate intestinal flora and address metabolic disorders associated with obesity and type 2 diabetes induced by high-fat diets (13, 14). However, the specific effects of APS on gut microbiota and metabolic characteristics in patients with MDD remain inadequately understood.

In addition to drugs, different diets are also associated with significant changes in gut microbiota (15), and studies have found that foods with probiotic activity can improve the abundance of gut microbiota in animals, stimulate the production of metabolites by certain gut microbes (16), and foods rich in GABA can improve neurological function (17). In addition to medicinal uses, *Astragalus* can also be developed as a functional food, and APS has become a new raw material for dietary supplements to strengthen the body’s immunity (18), with prebiotic effects (13). The newly developed *Astragalus* functional yogurt can be used as a special food for diabetics (18). Therefore, the development of APS as a functional food for healthy individuals has great potential, but the effect of APS on gut microbiota and metabolic characteristics of healthy individuals is still unclear.

Studies have proved that the microbiota fermented *in vitro* has a high similarity with the gut flora in the human body and is a useful tool for studying the interaction between drugs or functional factors and the human intestinal flora, with unique advantages such as ease of operation, low cost, and avoidance of host interference (19). At present, there are few data on *in vitro* fermentation of APS in MDD patients and healthy subjects, and the effects on the intestinal flora and metabolic characteristics are unclear. To this end, one of our study objectives is to use an *in vitro* fermentation model to elucidate the modulatory effect of APS on gut microbiota and metabolic profiles of MDD patients and healthy subjects by combining 16S sequencing technology and metabolomics. Another study objective is to explore the differences in gut microbiota and metabolic characteristics between MDD patients and healthy subjects, and to provide a basis for the development of drug therapies and functional foods.

## 2 Materials and methods

### 2.1 Study design and feces collection

The study protocol was approved by the Medical Ethics Committee of First Affiliated Hospital of Ningbo University on October 25, 2022 (No. 2022-046A-01), and registered at https://www.medicalresearch.org.cn (Identifier: MR-33-25-010733) on July 1, 2024. Patients with MDD were selected from in- and outpatients of the First Affiliated Hospital of Ningbo University, between July 2023 and December 2023. Subjects were eligible for this study if they (a) were currently experiencing a depressive episode according to the Diagnostic and Statistical Manual of Mental Disorders, Fifth Edition, as evidenced by a score of at least 17 on the 17-item Hamilton Depression Scale (HAMD-17); (b) aged 18–60 years; and (c) were experiencing their first acute episode without having taken any psychotropic medication before, such as antidepressants, anxiolytics, or antipsychotics.

Patients were excluded from this study if they (a) had a history of bipolar disorder, schizophrenia, or other mental disorders; (b) had a history of brain injury or surgery, or other serious dysfunctions of major organs; (c) had experienced alcohol or drug abuse; (d) had used probiotics, antidiarrheals, antibiotics, or other medications affecting the intestinal flora within four weeks prior to enrollment; (e) were pregnant or breastfeeding; (f) refused to take part in the study; and (g) had chronic diarrhea, constipation, or special dietary preferences (e.g., vegan, vegetarian).

Healthy controls were eligible for this study if they (a) scored less than seven on HAMD-17 and less than seven on the Hamilton Anxiety Scale (HAMA); (b) were aged 18–60 years; and (c) voluntarily participated in this study.

Healthy controls were excluded from this study if they (a) had experienced alcohol or drug abuse; (b) had used probiotics, antidiarrheals, antibiotics, or other medications affecting the intestinal flora in the four weeks before enrollment; (c) were pregnant or breastfeeding; and (d) had chronic diarrhea, constipation, or special dietary preferences.

Finally, a total of 15 patients with first-episode and drug-naïve MDD and 15 healthy subjects were recruited, and their general information and fecal samples were collected. All fecal samples were subjected to four different fermentation tests: the con group, the dep group, the APS-con group, and the APS-dep group. All included participants provided written informed consent.

### 2.2 *In vitro* fermentation

On enrollment morning, fasting participants provided fresh fecal samples (about 1 g) in 5-ml sterile tubes for immediate processing. A portion of these fresh fecal samples (0.6 g) was then combined with 6 mL of 0.1 M anaerobic phosphate-buffered saline (pH 7.0). Next, the feces were homogenized to obtain a 10% fecal suspension, which was filtered through sterile gauze to obtain the filtrate. Subsequently, 0.5 mL of filtrate was inoculated into 5 mL of sterilized medium and fermented in a 37 °C incubator for 48 h. Sterilize the medium at 121 °C for 30 min. The four fermentation groups were as follows: the con group (YCFA), the dep group (YCFA), the APS-con group (YCFA + 8 g/L APS), and the APS-dep group (YCFA + 8 g/L APS). Among them, APS was provided by Hengxing Pharmaceutical Research Institute (Hefei, China). The composition of the yeast extract–casein hydrolysate–fatty acid medium (YCFA) modified growth medium used in four fermentation groups were: Tryptone 10 g/l, Yeast extract 2.5 g/l, L-cysteine 1 g/l, NaCl 0.9 g/l, CaCl2·6H2O 0.009 g/l, KH2PO4 0.45 g/l, K2HPO4 0.45 g/l, MgSO4·7H2O 0.09 g/l, Hemoglobin 5 g/l, Resazurin 1 g/l, vitamin H (0.0005 g/l), vitamin B-12 (0.0005 g/l), vitamin B-6 (0.0075 g/l), p-aminobenzoic acid (0.0015 g/l), and folic acid (0.0025 g/l).

### 2.3 Determination of gas volume and composition

After 48 h of *in vitro* fermentation, the medium was collected. Then, the gases from the control medium (without inoculum) were tested to calibrate the analyzer. Finally, the gases in each processed medium were released into the gas analyzer (HL-QT01, Beiduokang High-Tech Co. Ltd., Hangzhou, China), and a gas sensor measured the gas production volume, including the total gas volume, CO_2_, and H_2_S production. The results were analyzed using the “MultiGas Analyzer.exe” software.

### 2.4 Gut microbiota analysis

After 48 h of fermentation, the 1.6 mL samples were centrifuged for 10 min (8, 000 g, 4 °C), the supernatant was removed, and the precipitated samples were stored in a refrigerator at -80 °C before being sent to Major Biotechnology in Shanghai, China, for 16S rRNA gene sequencing. The gut microbiota in the fecal samples was homogenized, and genomic DNA was extracted using the FastPure Stool DNA Isolation Kit (MJYH, Shanghai, China). The V3-V4 variable region fragment of the 16S rRNA gene was amplified by polymerase chain reaction (PCR), and the PCR amplification products were purified. Through the divisive amplicon denoising algorithm, ‘dereplication’ (equivalent to clustering at 100% similarity) was performed to obtain representative sequences with single-base accuracy, and amplicon sequence variants (ASVs) were used to construct a class operational taxonomic units table to obtain the final ASV feature table and feature sequences. Intestinal flora diversity analysis was performed using the VEGAN package in R software, with species classification and subsequent analysis conducted using the SILVA database (Release 138, https://www.arb-silva.de/documentation/release-138/) and the NT-16S database, applying an annotation threshold of >0.7 for confidence levels. Since all alpha diversity indices followed a normal distribution, we used repeated measures one-way analysis of variance (ANOVA) and Tukey’s *post-hoc* test for difference analysis. Using the QIIME2 and Vegan v2.5.3 packages, beta diversity was determined based on Bray-Curtis variability by Principal Coordinates Analysis (PCoA) and statistically assessed by ANOSIM analysis. The Kruskal-Wallis test was used to assess species differences based on ASV annotation results. To identify taxa with significant differences in relative abundance between groups, Linear Discriminant Analysis (LDA) Effect Size (LEfSe) software (LDA score > 4, p < 0.05) was used.

### 2.5 Targeted neurotransmitter metabolomic analysis

Before the analysis, the sample was then centrifuged at 14, 000 rpm (4°C, 10 min). Quantification of the species of interest in all samples was performed using ultra-performance liquid chromatography-tandem mass spectrometry (UHPLC–MS/MS) (ExionLC™ AD UHPLC-QTRAP 6500+, AB SCIEX Corp., Boston, MA, USA). A total of 8 GABA-related metabolites were detected. Waters HSS T3 column (2.1 × 100 mm) and two mobile phases (Phase A, 0.1% formic acid; Phase B, 0.1% formic acid in acetonitrile) were used for chromatographic separation by gradient elution. The column temperature was maintained at 35 °C, with an injection volume of 1 μL. The mobile phase flow rate was set to 0.30 mL/min, and the gradient program was set as follows: 0–1 min (0% B), 1–3 min (0–5% B), 3–5 min (5–10% B), 5–6 min (10–15% B), 6–7 min (15% B), 7–10 min (15–60% B), 10–11 min, (60–100% B), 11–12 min (100% B), 12–12.01 min, (100–0% B), 12.01–15 min, (0% B). Data were collected using a positive (negative) multiple response mode (MRM) pattern. The ion spray voltage was 5, 500 V (-4, 500 V), and the ion source temperature was set to 550 °C.

### 2.6 Statistical analysis

Statistical analysis was conducted using SPSS 28.0. Shapiro–Wilk one-sample test was used to confirm the normality. If continuous variables were normally distributed, one-way ANOVA was used to compare between-group differences; if variables were not normally distributed, the Mann-Whitney U test was used. The Chi-Square (χ^2^) test was utilized to assess the associations between categorical variables. Statistical values were expressed as mean ± standard deviation (`x ± s) for continuous variables and as numbers and percentages for categorical variables. The significance level was set at a two-tailed *p*-value of < 0.05.

## 3 Results

### 3.1 Participant characteristics

A total of 15 patients with MDD were recruited as the case group in our study. Concurrently, 15 healthy subjects undergoing physical examinations were chosen as healthy controls within the same timeframe. Notably, no significant differences were detected in demographic and clinical characteristics between the groups (*p* > 0.05) (**Table 1**).

**Table 1:** Demographic and clinical characteristics of participants.

Variables	Patients with MDD (n=15)	Healthy controls (n=15)	X^2^/t	*p*
Sex (Female/Male, n)	11/4	10/5	0.159	0.690
Age (y, `x ± s)	37.5 ± 12.6	31.3 ± 9.8	1.502	0.145
BMI (kg/m2, `x ± s)	22.9 ± 4.8	22.0 ± 2.6	0.638	0.530

### 3.2 Gas production during *in vitro* fermentation

**Figure 1:**
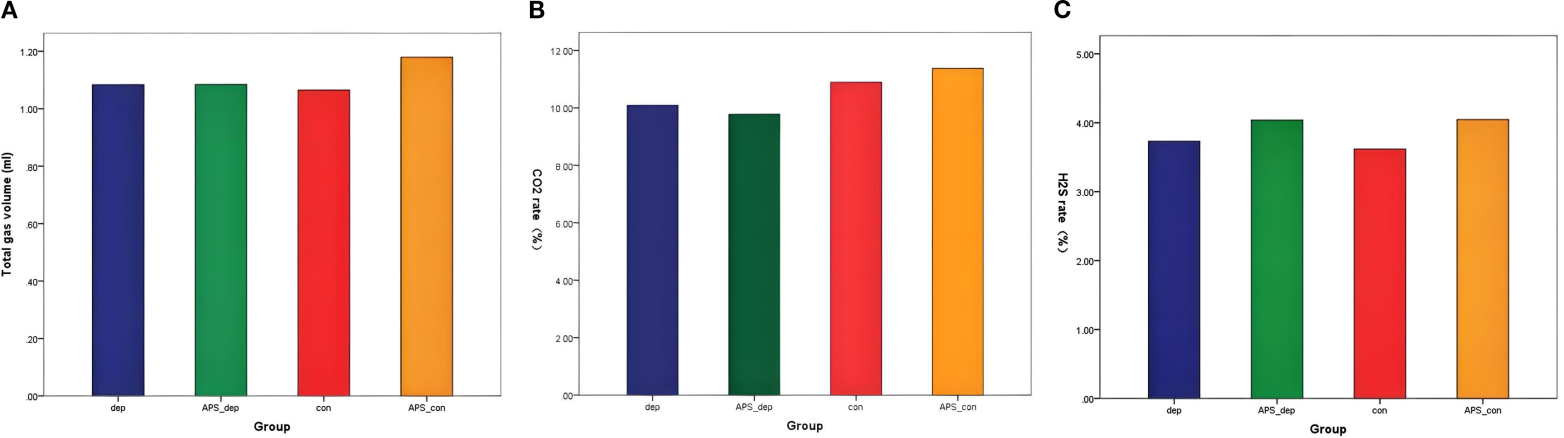
Effects of APS on gut gas in MDD patients and healthy subjects after *in vitro* fermentation. **(A)** Total gas production; **(B)** CO_2_ production ratio; **(C)** H_2_S production ratio.

As shown in **Figure 1**, there were no significant differences in the total volume of gas production, CO_2_, and H_2_S output among the con group, the APS-con group, the dep group, and the APS-dep group (all *p* > 0.05).

### 3.3 Comparison of gut microbiota diversity

**Figure 2:**
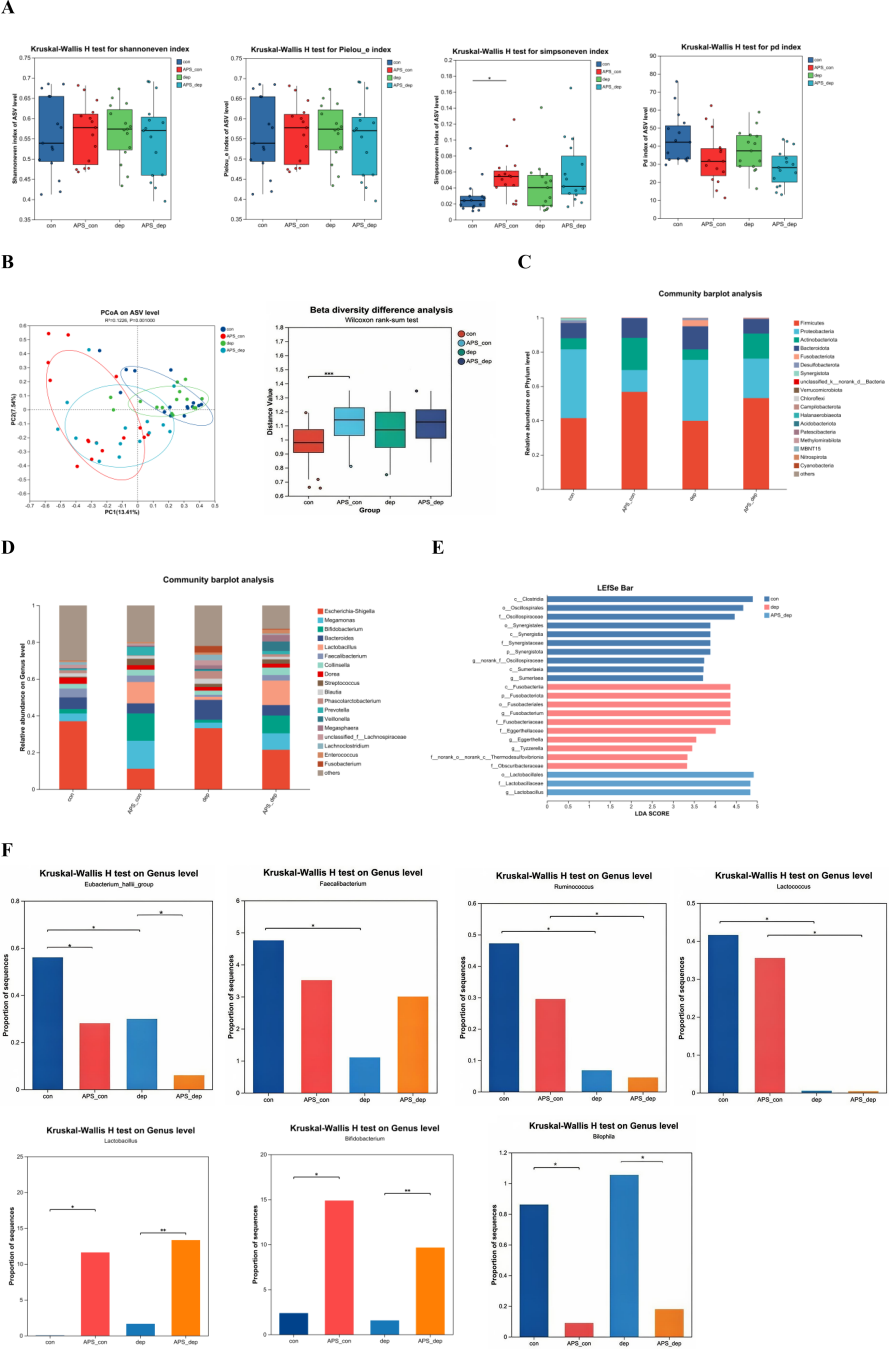
Effects of APS on the gut microbiota in MDD patients and healthy subjects after *in vitro* fermentation. **(A)** Shannoneven, Pielou, Simpsoneven and Pd indices; **(B)** principal Co-ordinates Analysis (PCoA) and Anosim Analysis; **(C)** the bacterial composition at the phylum levels; **(D)** the bacterial composition at the genus levels; **(E)** LEfSe analyses diagram (at the phylum to the genera levels); **(F)** Species-level composition analysis. The data differences are denoted as follows: ^*^0.01 < *p* ≤ 0.05, ^**^0.001 < *p* ≤ 0.01, and ^***^0.0001 < *p* ≤ 0.001.

Shannoneven, Pielou, Simpsoneven, and Pd indices represent alpha diversity of gut microbiota. The Simpsoneven index of the APS-con group was higher than that of the con group (*p* < 0.05), and there was no statistical difference in the other comparisons (all *p* > 0.05) (**Figure 2A**). The beta diversity was analyzed by Principal Coordinates Analysis (PCoA) and Anosim analysis. The results showed that the intestinal microbiota clustering and diversity were significantly higher in the APS-con group than that of the con group (*p* < 0.05), while there was no statistical difference in the other comparisons (all *p* > 0.05) (**Figure 2B**).

### 3.4 Species-level composition analysis

**Figures 2C, D** display the bacterial composition of four groups at the phylum and genus levels, respectively. LEfSe was used to better illustrate microbial communities with significant differences in abundance between groups, and LDA scores greater than 4 were considered indicative of significant differences in abundance between groups (*p* < 0.05). At the family level, *Oscillospiraceae* were the dominant bacteria in the con group, while *Fusobacteriaecae* and *Eggerthellaceae* were the dominant bacteria in the dep group. In contrast, *Lactobacillaceae* was the dominant bacteria in the APS-dep group (**Figure 2E**). At the genus level, the relative abundances of *Eubacterium hallii*, *Faecalibacterium*, and *Ruminococcus* in the con group were significantly higher than those in the dep group (*p* < 0.05) (**Figure 2F**). The relative abundances of *Bifidobacterium* and *Lactobacillus* were significantly higher in the APS-con group and APS-dep group compared with the con group and dep group (*p* < 0.05). Conversely, the relative abundances of *Eubacterium hallii* and *Bilophila* were significantly lower in the APS-con group and APS-dep group than that in the con group and dep group (*p* < 0.05). The relative abundance of *Lactococcus* and *Ruminococcus* in the APS-con group were significantly higher than those in the APS-dep group (*p* < 0.05).

### 3.5 Glu/GABA related metabolites

**Figure 3:**
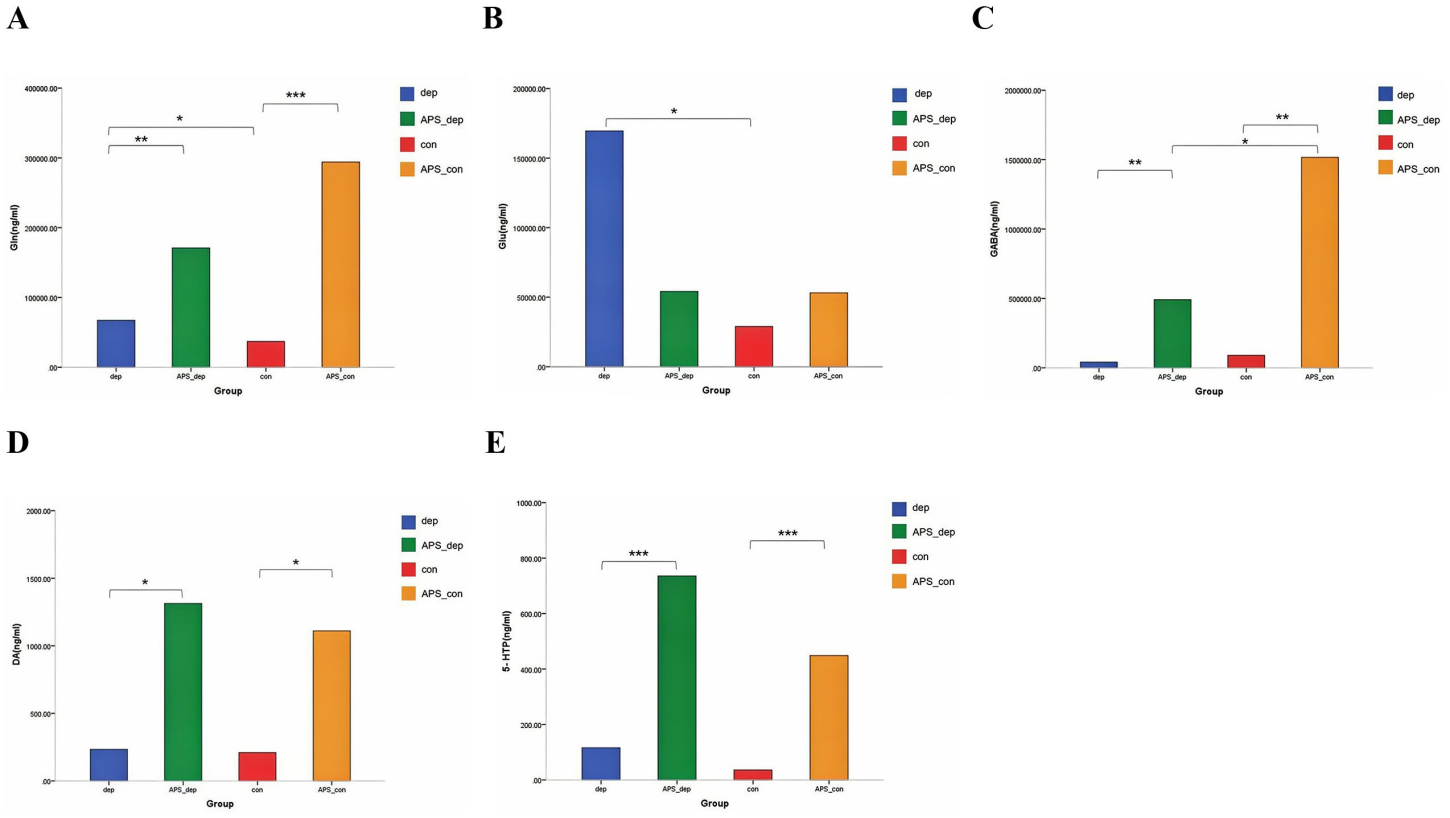
Effects of APS on Glu/GABA related metabolites in MDD patients and healthy subjects. **(A)** the levels of Gln; **(B)** the levels of Glu; **(C)** the levels of GABA; **(D)** the levels of DA; **(E)** the levels of 5-HTP. The data differences are denoted as follows: ^*^0.01 < *p* ≤ 0.05, ^**^0.001 < *p* ≤ 0.01, and ^***^0.0001 < *p* ≤ 0.001.

The examination of Glu/GABA-related metabolites revealed that the levels of Gln and Glu in the dep group were significantly higher those the con group (*p* < 0.05). The levels of Gln, GABA, DA, and 5-HTP were significantly higher in the APS-con group and APS-dep group compared with the con group and dep group (*p* < 0.05). In addition, the levels of GABA in the APS-dep group were significantly higher than those in the APS-con group (*p* < 0.05) (**Figure 3**).

## 4 Discussion

The brain-gut-microbiota axis (BGMA) denotes a bidirectional communication network involving the brain, gut, and gut microbiota. A growing body of evidence suggests that the gut microbiota plays a crucial role in modulating brain function and human behavior (20). Numerous observational studies have investigated the gut microbiota of patients with major depressive disorder (MDD), yet there remains a lack of consensus regarding specific differential species (21, 22). Generally, individuals with MDD exhibit higher relative abundances of pro-inflammatory species and lower relative abundances of short-chain fatty acid-producing bacteria (4). Several studies have demonstrated that bacteria such as *Eggerthellaceae* can disrupt the balance and integrity of the intestinal microecology. In contrast, bacteria such as *Lactobacillus*, *Bifidobacterium*, *Oscillospiraceae*, *Faecalibacterium*, and *Ruminococcus* may participate in the metabolism of various species within the body, potentially reducing the expression of inflammatory cytokines, enhancing the synthesis of short-chain fatty acids, and contributing to the integrity of intestinal mucosal structure and metabolic function (23–29). Our study employed *in vitro* fermentation models to analyze the gut microbiota and glutamate/gamma-aminobutyric acid (Glu/GABA)-related metabolites in both healthy subjects and patients with MDD. Following 48 hours of *in vitro* fermentation, a predominance of *Eggerthellaceae* was observed in MDD patients, whereas the abundance of *Eubacterium*, *Faecalibacterium*, and *Ruminococcus* was reduced compared to healthy subjects. Conversely, *Oscillospiraceae* was more prevalent in healthy subjects. These findings indicate an increase in the relative abundance of potentially pathogenic bacteria and a decrease in probiotics in the intestines of MDD patients, *aligning with* previous research (30). This dysbiosis of the intestinal microbiota may be linked to depressive episodes.

Furthermore, despite numerous prior investigations into the alterations in gut microbiota diversity among patients with MDD relative to healthy subjects, a definitive conclusion remains elusive. Ye et al. reported a significant reduction in the alpha diversity of gut microbiota in MDD patients compared to healthy controls (31). In contrast, Zhang et al. observed no difference in alpha diversity but noted variations in beta diversity (32). Shen et al. documented an increase in gut microbiota diversity among MDD patients (33), whereas Dong et al. found no differences between the two groups (34). The present study revealed no significant differences in either alpha or beta diversity between MDD patients and healthy subjects, aligning with the findings of Dong et al. (34). This discrepancy with other studies may be attributed to the *in vitro* fermentation approach employed, which effectively eliminated confounding factors such as host influence.

Previous research has demonstrated that gut microbiota significantly influence various critical host functions, including immune responses, endocrine and nervous systems, among others (35). The human gut microbiota may interact with the host brain via metabolites, facilitating bidirectional communication and playing a pivotal role in the pathogenesis of neuropsychiatric disorders (36). Recent studies have indicated that alterations in gut microbiota can affect Glu levels and the expression of Glu and GABA receptors in the brain (37, 38). Notably, Glu serves as the primary excitatory amino acid (EAA) and may function as a neurotransmitter in the gut (39), contributing significantly to neural damage (36). Preclinical studies have revealed that diets high in sodium Glu can induce depressive behaviors in rodent models (40, 41). Furthermore, research has identified plasma Glu levels as a potential indicator of disease severity in depression (39, 40), and alterations in glutamine (Gln) concentrations, a byproduct of Glu conversion, have also been observed in depressive conditions (40). Gut-derived Glu is also an important precursor raw material for Glu and GABA in the brain. GABA, as the most important inhibitory amino acid (IAA) in the brain, can inhibit neuronal excitation and reduce nerve cell damage caused by EAA, and has been shown to be closely related to mood disorders (42). It was found that the metabolic activity of GABA in the prefrontal cortex of depressed mice decreased, and Glu accumulated, resulting in excitatory neurotoxicity (43). Yan found that Glu levels increased and GABA levels decreased in patients with MDD (44). Therefore, the disruption of Glu/GABA homeostasis is crucial in the pathogenesis of depression (45). This study observed that Glu levels in patients with MDD were significantly elevated compared to healthy controls, aligning with previous research findings (44). However, no significant differences in GABA levels were detected, while Gln levels were increased. This observation may be related to the conversion processes between Glu and Gln, as well as the regulated supply of precursors necessary for GABA synthesis. The gut microbiota and its bioactive metabolites collaboratively maintain intestinal homeostasis and regulate systemic metabolism, thereby playing a crucial role in host health. Active metabolites identified in *in vitro* fermentation models may serve as indicators of the functional regulation of gut microbes, potentially via the gut-brain axis pathway, ultimately influencing the central nervous system.

The subsequent phase of this study investigated the impact of *Astragalus* polysaccharides (APS) on gut microbiota and GABA-related metabolites in patients with MDD and healthy subjects, utilizing *in vitro* fermentation models. *Astragalus*, a medicinal and edible homologous material, has been demonstrated to rectify imbalances in intestinal microbiota by significantly increasing the population of beneficial bacteria and restoring microbial diversity (46). In medicinal contexts, its primary component, APS, has exhibited notable antidepressant properties (12). Additionally, as a functional food, APS has emerged as a novel raw material for dietary supplements aimed at enhancing immune function (18) and has been recognized for its prebiotic effects (13). Nonetheless, the specific effects of APS on the gut microbiota and metabolic profiles of healthy subjects and MDD patients remain unclear. Current literature indicates that Bifidobacterium and Lactobacillus are probiotics known to modulate the gut microbiota, mitigate inflammation, and enhance the synthesis of short-chain fatty acids as well as the levels of monoamine neurotransmitters in both animal and human studies, which may contribute to the alleviation of depressive symptoms (47, 48). Conversely, *Bilophila*, recognized as an opportunistic pathogen, is capable of producing hydrogen sulfide (H_2_S), a compound potentially linked to the induction of intestinal inflammation and dysfunction of the intestinal barrier (49). In this study, APS was added to fecal samples from healthy subjects and MDD patients for *in vitro* fermentation, and the results showed that APS could increase the abundance of *Lactobacillus* and *Bifidobacterium* and reduce the abundance of *Bilophila* in both groups, indicating that APS may play a key role in increasing beneficial bacteria in the intestine, reducing harmful bacteria, and regulating intestinal flora for both MDD patients and healthy subjects, and has the property of medicinal and food homology. Subsequent analysis revealed that, in comparison to individuals with MDD, healthy subjects exhibited an increased abundance of *Lactococcus* and *Ruminococcus* following fermentation. Notably, *Lactococcus*, a pivotal strain in food fermentation, is known to reduce intestinal gas production, facilitate the colonization of probiotics such as *Bifidobacteria*, and inhibit both spoilage and pathogenic bacteria, thereby presenting promising applications in the food industry (50). *Ruminococcus* contributes to the synthesis of short-chain fatty acids, enhances metabolic processes, and supports normal intestinal function, particularly in healthy individuals, by improving receptor metabolism (51). Regarding species diversity, previous research has demonstrated that APS enhances the richness and diversity of the gut microbiota in cadmium-infected rats (13); however, its role in *in vitro* fermentation among healthy subjects and MDD patients remains underexplored. This study identified an increase in both alpha and beta diversity of APS post-fermentation in healthy subjects, whereas no significant changes were observed in MDD patients. The aforementioned results indicate that APS can better increase the diversity of intestinal flora and enhance the abundance of probiotics in healthy individuals, including *Lactobacillus*, *Bifidobacterium*, *Lactococcus*, and *Ruminococcus*. These microorganisms are crucial in modulating intestinal flora and improving metabolic processes. Consequently, APS holds substantial potential for the development of functional foods and for future applications in the prevention and treatment of depression.

Nevertheless, the consumption of prebiotics and probiotics can lead to increased gas production, which may adversely affect host health (52). Elevated levels of gas generated by gut microbiota can impede the extension of the colonic wall and alter the rate of material transit through the colon, potentially resulting in gastrointestinal symptoms (53). Carbon dioxide (CO_2_) has been identified as a significant by-product of carbohydrate fermentation by intestinal microbiota, which can modify the volume of the gut microenvironment and stimulate intestinal peristalsis (54). Additionally, the accumulation of hydrogen sulfide (H_2_S) is known to have detrimental health effects (55). In the present study, the incorporation of APS did not result in an increase in total gas volume, CO_2_, and H_2_S production, suggesting that APS supplementation did not induce significant gastrointestinal symptoms and may enhance adherence to dosing regimens.

Monoamine neurotransmitters, including dopamine (DA), 5-hydroxytryptamine (5-HT), norepinephrine (NE), and the homeostatic balance between glutamate (Glu) and gamma-aminobutyric acid (GABA), are typically identified as key targets in the mechanism of antidepressant effects (45). Studies have found that intestinal neurons and vagus nerve endings in the intestine can widely express a variety of neurotransmitter receptors, and GABA, DA, and 5-Hydroxytryptophan (5-HTP) produced by gut microorganisms can activate these receptors as signaling molecules, which in turn affect the function of the central nervous system through the vagus nerve and regulate mood and behavior (36–38). 5-HTP serves as a direct precursor to 5-HT, with the ability to traverse the blood-brain barrier and subsequently convert to 5-HT within the central nervous system. In the absence of 5-HTP, there may be an activation of the Glu system and inhibition of the GABA system, resulting in an imbalance between Glu and GABA, which is potentially linked to the pathogenesis and progression of depression (56). For many years, it was believed that GABA was synthesized in the brain, and that exogenous GABA could not traverse the blood-brain barrier to augment its cerebral concentration. However, recent studies have demonstrated that GABA can access the peripheral nervous system of the brain, including regions such as the perihypothalamic area, thereby influencing mood and behavior. Additionally, GABA is produced in the mammalian intestine and its synthesis is modulated by various bodily systems (57). GABA contributes to gastrointestinal health by regulating the intestinal microbiota, and alterations in the microbiota can, in turn, affect intestinal GABA levels (58). Experiments utilizing germ-free mice, which were categorized into germ-free and normal microbiota-transplanted groups, revealed that GABA concentrations in the feces and blood of germ-free mice were significantly lower than those in mice with transplanted normal flora (59). These findings indicate that GABA is not only synthesized in the gut of mice but that the gut microenvironment also plays a crucial role in modulating its production. Supplementation with GABA and Gln has been shown to enhance the gut microenvironment, elevate levels of GABA and 5-HT, and modulate the central nervous system’s functions (57). Chen et al. demonstrated that light tempeh enriched with GABA could ameliorate depression-like behaviors in mice while simultaneously increasing levels of 5-HT and DA, with these effects being correlated with the GABA content (60). Additionally, Qu et al. reported that *Bifidobacteria* and *Lactobacillus* can ferment to produce substantial quantities of GABA, and that probiotics capable of GABA production may alleviate symptoms associated with depression (8). Although it has been established that APS possesses antidepressant properties, and this study also indicates that APS can enhance the abundance of *Bifidobacterium* and *Lactobacillus*, the impact of APS on Glu/GABA metabolism remains to be elucidated. This study observed that after adding APS, the levels of Gln, GABA, DA, and 5-HTP in *in vitro* fermentation samples from patients with MDD increased, but there was no statistically significant difference in Glu, which may be related to altered metabolic function of gut microbes, and the high content of GABA contained in astragalus itself (61). This suggests that the addition of APS may indirectly promotes the increase of these metabolites and neurotransmitters, and by regulating the interaction between gut microbiota and bioactive metabolites, it may ultimately exert an antidepressant effect in the central nervous system through the gut-brain axis pathway. Regulating GABA metabolism may become a new target for antidepressant therapy. However, the amount of GABA in the human body decreases with age, so it is important to supplement GABA in daily life to improve health (62). Functional foods developed based on GABA’s efficacy are believed to prevent or improve neurological function (17), and *Astragalus* is noted for its high GABA content (61). The study also observed that, compared to MDD patients, APS fermentation resulted in higher GABA levels in healthy subjects, indicating that APS may be more effective in modulating GABA in healthy individuals and could serve as a promising ingredient for future health food products.

To our knowledge, this is an earlier study to use an *in vitro* fermentation model to elucidate the effects of APS on the gut microbiota and Glu/GABA-related metabolites in MDD patients and healthy subjects, and to identify changes in key bacterial species that are strongly associated with APS treatment. This may also be the first study to explore the role of APS in healthy subjects and whether it can be used as a functional food for development. In addition, since we studied first-onset and drug-naïve MDD patients, the influence of confounding factors such as medication and episode frequency was ruled out. However, this study has some limitations. Firstly, the small sample sizes, population characteristics, lifestyle and dietary habits may result in varying research outcomes. To mitigate these impacts, we consulted sample size estimates from established *in vitro* fermentation models prior to conducting the study (63, 64). Subsequent analysis revealed no statistically significant differences between groups in key variables, including age, sex, and BMI. Furthermore, the *in vitro* fermentation model highlighted the interactions between APS and microbial communities, thereby mitigating the interference from more complex confounding factors present in the *in vivo* study to a certain extent. Secondly, *in vitro* experiments may not fully replicate *in vivo* conditions, leading to potential discrepancies between results obtained from *in vitro* fermentation and those derived from the actual environment of the human gut. In this study, APS was directly added to fecal suspension for fermentation, omitting the pre-digestion step typically employed in *in vivo* experiments. This methodology may underestimate the benefits of intact polysaccharides; however, it effectively minimizes the influence of complex variables such as individual differences in digestibility, variations in absorption, and gastric emptying time. As a result, this approach enabled a direct assessment of the net effects of APS on gut microbiota and metabolites, thereby establishing a clear and controlled baseline environment for elucidating the fundamental mechanisms underlying APS interactions with microorganisms. Although this study cannot definitively confirm the magnitude of APS benefits, the role demonstrated by APS in the *in vitro* model indicates its inherent potential for utilization by gut microbes. The positive *in vitro* results suggest a high likelihood of activity *in vivo*, which can be further explored through subsequent multi-stage *in vitro* models and *in vivo* experiments. This lays a preliminary foundation for progressively unveiling the comprehensive role of APS in the entire physiological process. Finally, there is a failure to adequately elucidate the coherent biological mechanisms underlying microbial and metabolite changes and to integrate them with known gut-brain axis pathways. Future research should aim to develop more comprehensive gut-brain axis models to elucidate the interactions between APS-induced microbial alterations and neuroactive metabolites, along with their underlying mechanisms. Further validation of the findings from this study through animal and clinical experiments is essential. Despite its limitations, our study contributes valuable insights into the potential application of APS in the prevention and treatment of depression, as well as in the development of functional foods. Future research will focus on identifying therapeutic targets among gut microbes and related metabolites for neurological and psychiatric disorders, alongside the development and application of APS in food and pharmaceuticals.

## 5 Conclusion

The results showed that after 48 h of *in vitro* fermentation, the composition of the intestinal microbiota of MDD patients was significantly changed compared with the healthy subjects, which was characterized by an increase in pathogenic bacteria and a decrease in probiotics, mainly manifested as elevated *Fusobacteriaecae* and *Eggerthellaceae*, and reduced levels of *Eubacterium hallii*, *Faecalibacterium*, and *Ruminococcus*. Elevated levels of Glu and Gln in MDD patients may serve as markers for the regulation of gut microbial function, suggesting that increased excitatory amino acid neurotransmitters and their precursors could be associated with depressive episodes via the gut-brain axis. APS addition was shown to improve gut microbial dysbiosis by increasing the abundance of probiotics, mainly *Lactobacillus* and *Bifidobacterium*, while decreasing the abundance of opportunistic pathogenic bacteria, such as *Bilophila*, without increasing the total gas volume, CO_2_ and H_2_S production and causing flatulence. In healthy subjects, the diversity of intestinal flora increased after the addition of APS was observed, and the increase of probiotics, including *Lactobacillus*, *Bifidobacterium*, *Lactococcus* and *Ruminococcus*. Furthermore, beneficial metabolites were elevated in MDD samples after APS addition, including Gln, GABA, DA, and 5-HTP. The increase in GABA among healthy subjects was more significant. This suggests that the addition of APS may indirectly promote the increase of key metabolites in the gut in the *in vitro* fermentation model. Based on the findings derived from the *in vitro* fermentation model, we propose hypotheses that can be verified *in vivo* studies: MDD is associated with disruptions in gut microbiota and Glu/GABA metabolism. When APS are applied directly to the colon, they may mitigate these disruptions by modulating microbial composition and key neuroactive metabolites, thereby influencing the central nervous system via the gut-brain axis. This effect appears to be more pronounced in healthy individuals. These findings suggest that APS holds potential value for the prevention and treatment of depression, as well as for the development of functional foods. Future randomized controlled trials should consider focusing on these specific endpoints.

## Data Availability

The raw data supporting the conclusions of this article will be made available by the authors, without undue reservation.
